# Conjugation of DM1 to anti-CD30 antibody has potential antitumor activity in CD30-positive hematological malignancies with lower systemic toxicity

**DOI:** 10.1080/19420862.2019.1618674

**Published:** 2019-06-04

**Authors:** Yijun Shen, Tong Yang, Xuemei Cao, Yifan Zhang, Li Zhao, Hua Li, Teng Zhao, Jun Xu, Hengbin Zhang, Qingsong Guo, Junli Cai, Bei Gao, Helin Yu, Sicheng Yin, Ruiwen Song, Jingsong Wu, Lingyu Guan, Guanghao Wu, Li Jin, Yong Su, Yanjun Liu

**Affiliations:** a Ministry of Education Key Laboratory of Contemporary Anthropology, Fudan University, Shanghai, China; b R&D Department of Genetic Engineering, Shanghai Fudan-Zhangjiang Bio-Pharmaceutical Co., Ltd., Shanghai, China; c Department of Technical Quality, Shanghai Jiaolian Drug Research and Development Co., Ltd, Shanghai, China

**Keywords:** Antibody drug conjugate (ADC), hematological malignancies, CD30, DM1, Hodgkin’s disease, anaplastic large cell lymphoma, cutaneous T-cell lymphoma, bystander effect

## Abstract

An anti-CD30 antibody-drug conjugate incorporating the antimitotic agent DM1 and a stable SMCC linker, anti-CD30-MCC-DM1, was generated as a new antitumor drug candidate for CD30-positive hematological malignancies. Here, the *in vitro* and *in vivo* pharmacologic activities of anti-CD30-MCC-DM1 (also known as F0002-ADC) were evaluated and compared with ADCETRIS (brentuximab vedotin). Pharmacokinetics (PK) and the safety profiles in cynomolgus monkeys were assessed. Anti-CD30-MCC-DM1 was effective in *in vitro* cell death assays using CD30-positive lymphoma cell lines. We studied the properties of anti-CD30-MCC-DM1, including binding, internalization, drug release and actions. Unlike ADCETRIS, anti-CD30-MCC-DM1 did not cause a bystander effect in this study. *In vivo*, anti-CD30-MCC-DM1 was found to be capable of inducing tumor regression in subcutaneous inoculation of Karpas 299 (anaplastic large cell lymphoma), HH (cutaneous T-cell lymphoma) and L428 (Hodgkin’s disease) cell models. The half-lives of 4 mg/kg and 12 mg/kg anti-CD30-MCC-DM1 were about 5 days in cynomolgus monkeys, and the tolerated dose was 30 mg/kg in non-human primates, supporting the tolerance of anti-CD30-MCC-DM1 in humans. These results suggest that anti-CD30-MCC-DM1 presents efficacy, safety and PK profiles that support its use as a valuable treatment for CD30-positive hematological malignancies.

## Introduction

Antibody-drug conjugates (ADCs), which are composed of potent cytotoxic drugs linked to antibodies via chemical linkers, allow specific targeting of drugs to neoplastic cells.^1–5^ These powerful anti-cancer agents are designed to allow specific targeting of highly potent cytotoxic agents to tumor cells while sparing healthy tissues. Four ADCs have received approval from the Food and Drug Administration (FDA) and the European Medicines Agency, and can be prescribed for metastatic conditions, while around 60 ADCs are currently enrolled in clinical trials.^6^

ADCETRIS® (brentuximab vedotin) is a CD30-directed ADC indicated for the treatment of patients with relapsed or refractory Hodgkin’s lymphoma (HL), systemic anaplastic large cell lymphoma (ALCL) and cutaneous T-cell lymphoma (CTCL) who have received prior systemic therapy.^7–10^ HL is characterized by the presence of Hodgkin and Reed-Sternberg (HRS) cells, which comprise only a minority of cells in the tumor mass and express CD30 surface antigen. CD30 is also expressed on subsets of non-Hodgkin’s lymphoma, including ALCL and CTCL, and on rare solid tumors such as germ-line malignancies.^7^ ADCETRIS® consists of three components: (1) the chimeric antibody cAC10, specific for human CD30; (2) the potent antimicrotubule agent monomethyl auristatin E (MMAE); and (3) a protease-cleavable linker that covalently attaches MMAE to cAC10.^11–13^ The biological activity of ADCETRIS results from a multistep process. Binding of the ADC to CD30 on the cell surface initiates internalization of the ADC-CD30 complex, which then traffics to the lysosomal compartment. Within the cell, a single defined active species, MMAE, is released via proteolytic cleavage. Binding of MMAE to tubulin disrupts the microtubule network within the cell, induces cell cycle arrest, and results in apoptotic death of the CD30-expressing tumor cell.^11^

Treatment of the mixtures of CD30-positive (HL cell line L540cy and ALCL cell line Karpas 299) and CD30-negative cell lines with ADCETRIS® demonstrated that diffusible released MMAE from CD30-positive cells was able to kill cocultivated CD30-negative cells. These evidence indicates a ‘bystander effect’ of ADCETRIS®, whereby free MMAE is released from dying cells in concentrations sufficient to kill neighboring tumor cells either directly or indirectly by altering the tumor microenvironment.^14^ This bystander effect might explain the “paradoxical lack of correlation” existing between tumor CD30 expression and clinical response to ADCETRIS®.^15^ Although bystander effects of targeted therapy that overcome heterogeneous expression of the target antigen might have important clinical consequences, the free MMAE released to the system also elicits side effects. The most common adverse reactions in at least 20% of patients treated with ADCETRIS® across all clinical trials were neutropenia, anemia, peripheral sensory neuropathy, nausea, fatigue, constipation, diarrhea, vomiting, and pyrexia.^16^ On January 13, 2012, the FDA announced that because ADCETRIS® had been linked with two cases of progressive multifocal leukoencephalopathy, the administration was requiring the addition of a black box warning to the drug label regarding this potential risk.^17^ In other respects, MMAE resistance is considered one of the possible modes of ADCETRIS® resistance for Hodgkin’s lymphoma both *in vitro* and in patients. Chen et al. proposed that drug transporters can play a role in ADCETRIS® drug resistance in Hodgkin’s lymphoma, they found the HL cell line L428, but not the ALCL cell line Karpas 299, exhibited MMAE resistance and increased expression of the MDR1 drug exporter compared with the parental line.^18^ Although MMAE can be actively pumped out of the cell by *P*-glycoprotein or other transporters, there are other cytotoxic agents that can be linked to ADCs that are not substrates for transport, and these could be utilized as CD30 immunoconjugates in the future.

In order to improve the safety of ADCETRIS®, we generated anti-CD30-MCC-DM1 (also known as F0002-ADC), consisting of the anti-CD30 antibody (cAC10) chemically conjugated to the antimitotic agent maytansinoid *N*(2ʹ)-deacetyl-*N*(2ʹ)-(3-mercapto-1-oxopropyl)-maytansine (DM1) with a stable linker: succinimidyl trans-4-(maleimidylmethyl)cyclohexane-1-carboxylate (SMCC). The stable linker ADC approach has been successfully applied to clinical treatment. Kadcyla® (ado trastuzumab emtansine, T-DM1, anti-HER2-MCC-DM1) shows encouraging efficacy and safety in the treatment of HER2-positive breast cancer.^19,20^ Furthermore, replacing vc-MMAE with MCC-DM1 may help to overcome resistance. Here, we report that anti-CD30-MCC-DM1 specifically binds to, internalizes within, and exhibits cytotoxicity against CD30-expressing cell lines *in vitro*. We investigated the efficacy, and safety of anti-CD30-MCC-DM1 in animal models to assess its potential as a therapeutic for the treatment of CD30-positive lymphomas, including L428, Karpas 299 and HH cell lines, which are derived from HL, ALCL, and CTCL, respectively. It was found that anti-CD30-MCC-DM1 can induce sustained tumor regression in xenograft mouse models. Meanwhile, the tolerance dose of anti-CD30-MCC-DM1 was higher than that of ADCETRIS®, and it was presumed that the released drug Lys-MCC-DM1 has no bystander effect. Based on these promising data, a Phase 1 study (NCT03894150) of anti-CD30-MCC-DM1 is currently enrolling patients in China.

## Results

### Generation and characterization of anti-CD30-MCC-DM1

**Figure 1.**
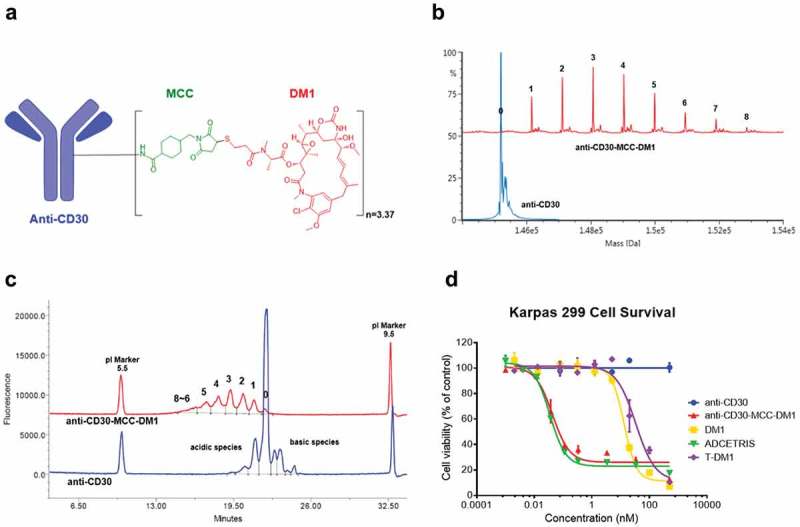
Structure and characterizations of anti-CD30-MCC-DM1. a, Structure of anti-CD30-MCC-DM1. b, Deconvoluted intact mass spectra of anti-CD30 mAb and anti-CD30-MCC-DM1 acquired by LC-MS; all samples were treated with PNGase F. c, Electrophoretogram of anti-CD30 mAb and anti-CD30-MCC-DM1 acquired by imaged capillary isoelectric focusing. d, *In vitro* sensitivity of Karpas 299 cells to anti-CD30 mAb and ADCs. Karpas 299 cells were plated at 5000 cells/well and were exposed to a gradient titration of anti-CD30 (the parental mAb cAC10); anti-CD30-MCC-DM1; DM1 (the free drug); ADCETRIS (anti-CD30-vc-MMAE); or T-DM1 (a control ADC (anti-HER2-MCC-DM1)). Cells were assessed for cytotoxicity by the Alamar Blue assay after 96 h of continuous exposure, as described in the “Materials and methods.” The percentage cell viability was relative to untreated control wells. Results for each study are plotted as the mean (± SEM).

Conjugates of DM1 were formed using the chimeric monoclonal antibody (mAb) cAC10,^21^ recognizing the CD30 antigen, through the uncleavable linker SMCC on lysine residues. The resulting conjugate, anti-CD30-MCC-DM1 with drug-to-antibody ratio (DAR) 3.37 (Figure 1(a)), is referred to as anti-CD30-MCC-DM1 in this study. Since anti-CD30 antibodies have numerous surface accessible lysine residues that can potentially react with the sulfhydryl of the DM1 drug through the SMCC linker, anti-CD30-MCC-DM1 is a mixture of conjugated species. The distribution of the payload was determined by intact mAb liquid chromatography (LC)/tandem mass spectrometry (MS) (after PNGase F treatment to remove N-glycans) (Figure 1(b)) and imaged capillary isoelectric focusing (iCIEF) (Figure 1(c)). The results acquired by both methods show similar distributions and DAR values. (MS-DAR is 3.37 and iCIEF-DAR is 3.35).

To evaluate the *in vitro* specific cytotoxicity of anti-CD30-MCC-DM1, CD30-positive Karpas 299 cells were exposed to anti-CD30 (the parental mAb cAC10); anti-CD30-MCC-DM1; DM1 (the free drug); ADCETRIS (anti-CD30-vc-MMAE); or T-DM1 (a control ADC (anti-HER2-MCC-DM1)). Figure 1(d) shows that anti-CD30-MCC-DM1 was potently cytotoxic to Karpas 299 cells, with an IC_50_ value of 0.06 nmol/L, which was comparable with ADCETRIS (0.04 nmol/L). In contrast, the IC_50_ of T-DM1 was 31.02 nmol/L; thus, the nonbinding control ADC was 500-fold less cytotoxic than anti-CD30 ADCs. Under these conditions, unconjugated mAb had no effect on cytotoxicity.

### In vitro potency of anti-CD30-MCC-DM1

The cytotoxicity and selectivity of anti-CD30-MCC-DM1 were evaluated compared with free drug DM1 on a panel of CD30-positive and CD30-negative cell lines. Surface expression of CD30 on the various lines was quantified (Table 1); cells with CD30 levels below 100 molecules per cell were considered negative for CD30 expression. All of the antigen-positive cell lines were highly sensitive to anti-CD30-MCC-DM1 cytotoxicity, with IC_50_ values below 0.13 nmol/L (ranging from 0.05 to 0.13 nmol/L). In contrast to CD30-positive cells, the antigen-negative lines were insensitive to anti-CD30-MCC-DM1. There was no significant selectivity in sensitivity to DM1 between CD30-positive and negative cell lines (Supplementary Fig. S1), with IC_50_ values ranging from 7.06 to 39.53 nmol/L. In CD30-positive cells, the ADC with equivalent DM1 was found to be more active than free drug, suggesting that selective sensitivity was conferred by the ADC.

**Table 1.** Cytotoxicity of anti-CD30-MCC-DM1 and free DM1 on lymphoma lines.

Cell line	Lineage	No. of surface CD30 molecules per cell	IC_50_ (nmol/L DM1 equivalents)	
			Anti-CD30-MCC-DM1	DM1
HH	Cutaneous T-cell lymphoma	134,431	0.05 ± 0.012（*n* = 3）	7.06 ± 2.44（*n* = 4）
Karpas 299	Anaplastic large cell lymphoma	217,234	0.12 ± 0.012（*n* = 3）	21.26 ± 9.06（*n* = 4）
SU-DHL-1	Anaplastic large cell lymphoma	52,597	0.13 ± 0.012（*n* = 4）	9.92 ± 2.70（*n* = 4）
L540	Hodgkin’s disease, T-cell like	414,959	0.13 ± 0.009（*n* = 3）	39.53 ± 1.47（*n* = 3）
L428	Hodgkin’s disease, B-cell like	56,494	0.07 ± 0.014（*n* = 4）	7.84 ± 2.57（*n* = 4）
Raji	Burkitt lymphoma B cell	0	N/A（*n* = 3）	30.18 ± 0.50（*n* = 3）
Ramos	Burkitt lymphoma B cell	50	N/A（*n* = 3）	11.94 ± 1.84（*n* = 6）

IC_50_ values were determined from four-parameter curve fitting and are expressed as the mean ± SEM. The DM1 equivalent molar concentration of ADC was calculated by multiplying the concentration of ADC by its DAR N/A = not applicable, which means the complete four-parameter curve is not available.

### Binding, internalization, and drug release of anti-CD30-MCC-DM1

**Figure 2.**
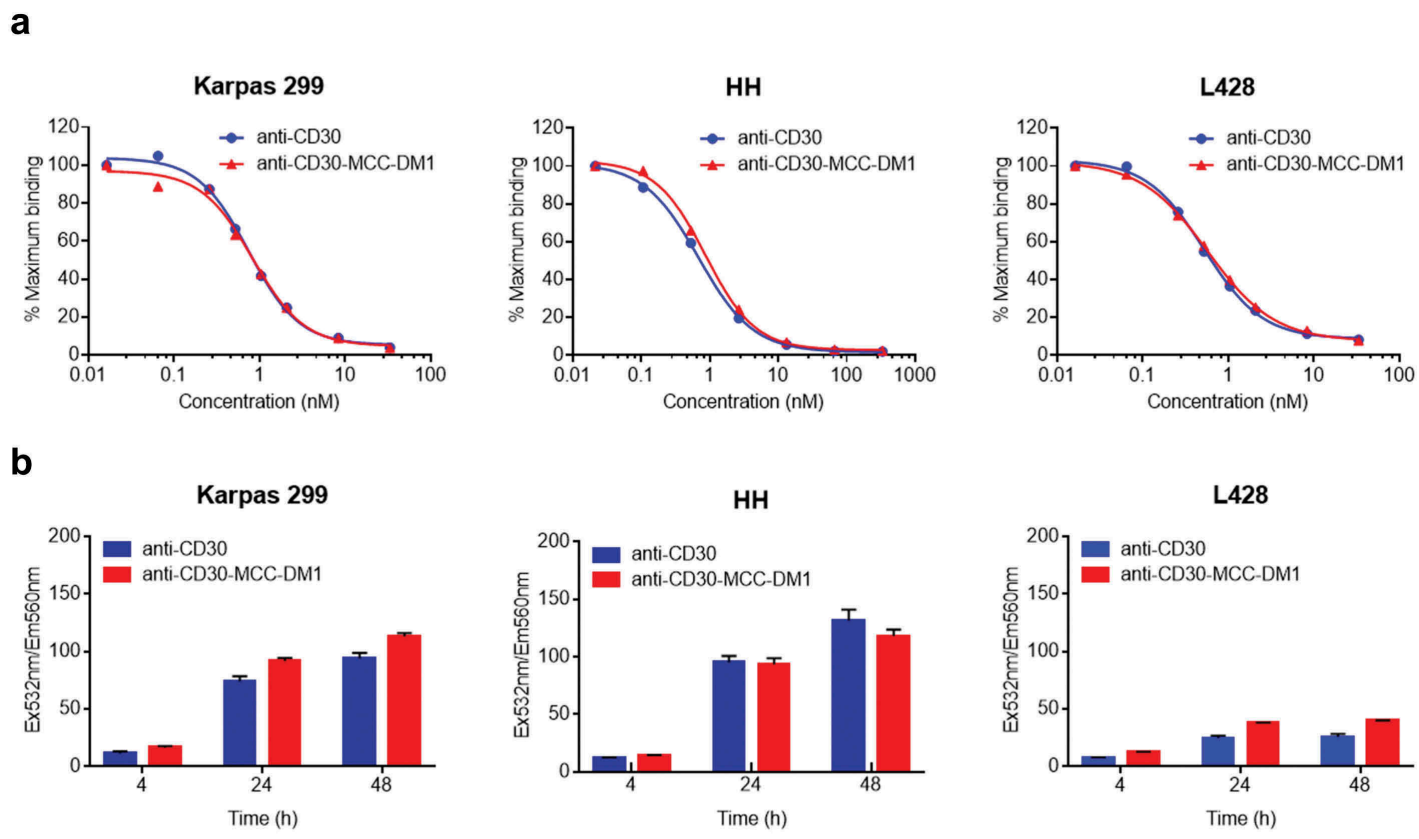
Binding and internalization of anti-CD30-MCC-DM1. a, Competition binding of anti-CD30-MCC-DM1 to CD30-positive cells. Karpas 299, HH and L428 cells were combined with biotinylated anti-CD30 mAb and serial dilutions of either anti-CD30 mAb or anti-CD30-MCC-DM1. The normalized fluorescence intensities were plotted versus mAb concentration as described in the “Materials and methods.” b, internalization of anti-CD30-MCC-DM1 into CD30-positive cells. pHAb dye-conjugated anti-CD30 mAb and anti-CD30-MCC-DM1 were added to the Karpas 299, HH, and L428 cells and incubated for various time durations. Mean and standard deviations from triplicate readings are plotted.

ALCL, HL and CTCL are approved indications of ADCETRIS. Karpas 299 (ALCL), HH (CTCL) and L428 (HL) were measured to contain relatively high, medium and low expression of CD30. They were chosen for the multistep process studies of anti-CD30-MCC-DM1. First, competition binding experiments were performed to determine whether conjugation of DM1 to the anti-CD30 mAb interferes with the CD30 binding capability of the ADCs. Karpas-299, HH, and L428 cells were incubated with biotinylated anti-CD30. Anti-CD30-MCC-DM1 effectively competed with biotin-labeled anti-CD30 mAb equivalently to unlabeled anti-CD30 mAb, as shown in Figure 2(a). Thus, conjugation with DM1 did not reduce the CD30 binding capabilities of the ADCs.

For CD30-mediated ADC internalization studies, anti-CD30 mAb and anti-CD30-MCC-DM1 were conjugated with pHAb dye and incubated with cells expressing CD30. Upon binding to the receptor, the dyes conjugated to the antibody are not fluorescent because of the neutral pH of the media; however, upon internalization, the pH decreases and the dyes become fluorescent.^22^ Internalization of anti-CD30 mAb and anti-CD30-MCC-DM1 could be detected within 4 h, fluorescence signal increased at 24 h, and the signal approached saturation at 48 h. Internalization was specific: no signal was observed when these antibodies were incubated with CD30-negative Ramos cells (Supplementary Fig. S2). Absolute fluorescence signals in L428 cells at 24 and 48 h were lower compared to the detected absolute fluorescence signals in Karpas 299 and HH, which was correlated with an observed lower CD30 expression level of L428 than the ones of Karpas 299 and HH. (Figure 2(b)).

**Figure 3.**
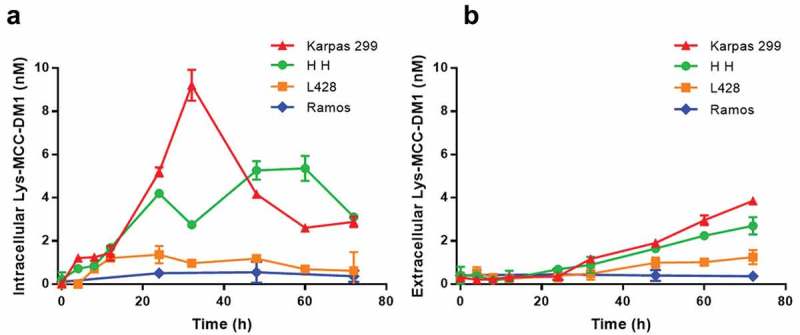
Generation of free drug in cells treated with anti-CD30-MCC-DM1. Intracellular (a) and extracellular (b) concentrations of Lys-MCC-DM1 from 0 to 72 h of exposure with anti-CD30-MCC-DM1 small molecule were determined with an LC-MS/MS method in CD30-positive (Karpas 299, HH, and L428) and CD30-negative (Ramos) cell cultures. Results for each test are plotted as the mean (± SEM).

The contents of intracellular and extracellular released drug were determined with an LC-MS/MS method that simultaneously detects potential catabolites of the anti-CD30-MCC-DM1.The three CD30-positive cells and a negative cell line were incubated with 400 ng/ml ADC for 4, 8, 12, 24, 43, 48, 60, and 72 h. As shown in Figure 3, Ramos cells are CD30-negative, and released drug was only detected at the lower limit of quantitation (LLOQ) level that can be neglected through 3 days of the assay. The three CD30-positive cell lines all generated released drug. The intracellular drug concentrations of Karpas 299 cells maintained a relatively high level in the first 12 h, increased rapidly at 24 and 32 h, and then decreased over the next hours. HH cell intracellular concentrations were lower in the first 36 h and higher during 48 h to 72 h compared to the case of Karpas 299 cells. The intracellular concentrations in L428 cells were only slightly higher than in CD30-negative cells and remained at low levels throughout the assay. The extracellular release of Lys-MCC-DM1 increased in a time-dependent and antigen expression level-relevant manner. Concentrations of intracellular and extracellular MCC-DM1 and DM1 were determined simultaneously, and all quantitation results were below or near the LLOQ (data not shown). It seems that Lys-MCC-DM1 is the major catabolite mediated by the lysosomes in the targeted cells, which is consistent with the catabolic fate of T-DM1.^23^

### Cell cycle effects and bystander activity of anti-CD30-MCC-DM1

**Figure 4.**
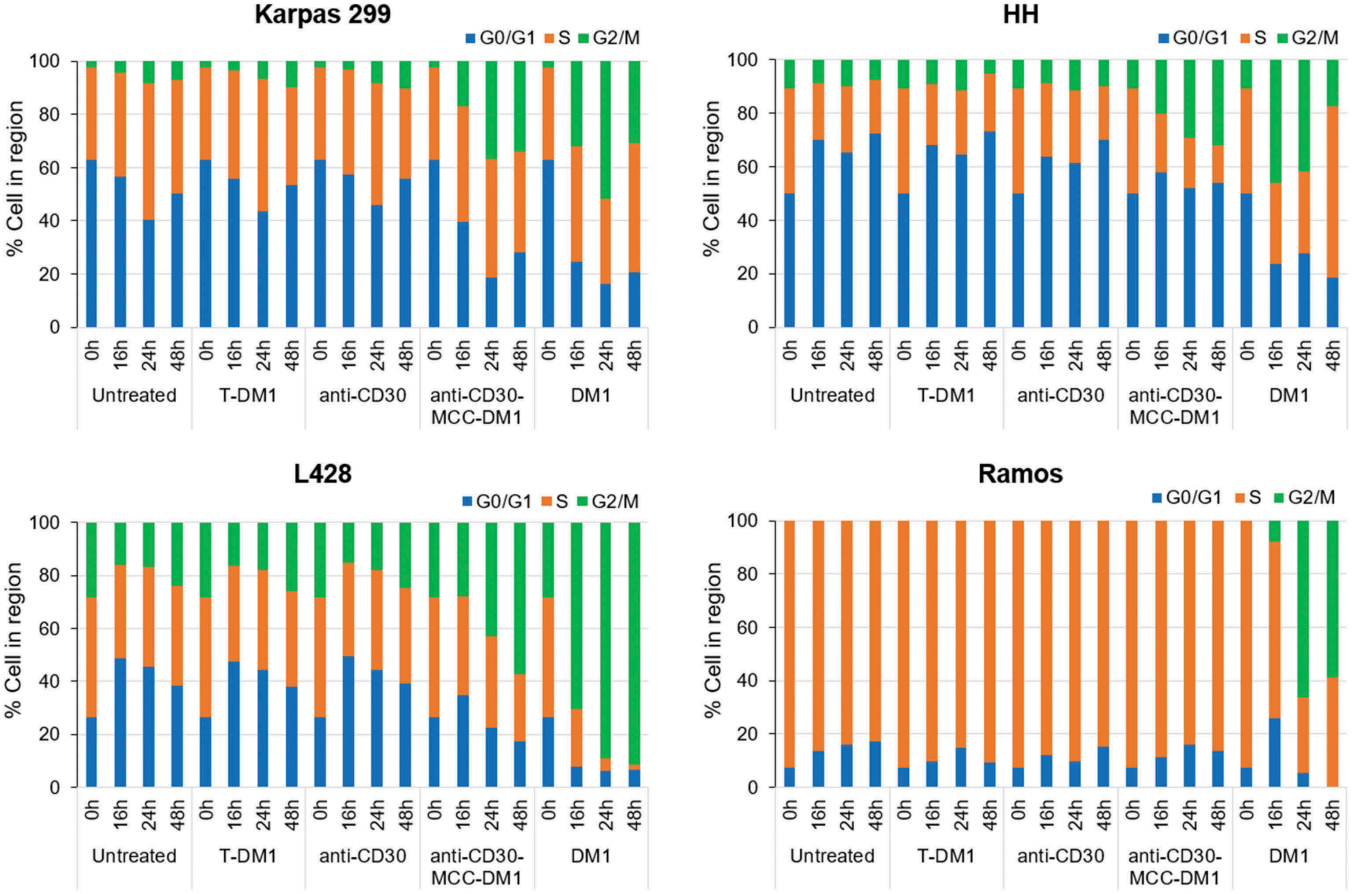
Effects of mAb, ADC and DM1 on the cell cycle. CD30-positive Karpas 299, HH, and L428 and CD30-negative Ramos cells were untreated or treated with anti-CD30 mAb, anti-CD30-MCC-DM1, control ADC T-DM1, or DM1 (an equimolar amount to anti-CD30-MCC-DM1) and incubated for 0, 16, 24, or 48 h. At each time point, the cells were labeled with propidium iodide, and the cell cycle position was measured by flow cytometry.

The effects of anti-CD30-MCC-DM1 on the cell cycle were examined using CD30-positive Karpas 299, HH and L428 cells, and CD30-negative Ramos cells. The equivalent antibody or small molecule molar concentrations of anti-CD30, T-DM1, and DM1 were used as comparators. In CD30-positive cells, treatment with anti-CD30 mAb or the irrelevant ADC T-DM1 showed no significant difference in cell cycle position compared with untreated control cells. Karpas 299 and HH cells exposed to an equal level of anti-CD30-MCC-DM1 showed a significant increase in G2/M phase cells and coincident loss of G0/G1 phase cells within 16 h of exposure, while 24 h of exposure to L428 caused an increase in G2/M phase cells (Figure 4); these effects were consistent with G2/M-phase arrest induced by DM1. In contrast, anti-CD30-MCC-DM1 displayed no effect on Ramos cells despite the finding that DM1 significantly induced G2/M-phase arrest in these cells. These data suggest that anti-CD30-MCC-DM1 selectively induced growth arrest in G2/M phase in CD30-positive cells, and that this effect was not seen after sustained exposure to equivalently high concentrations of control T-DM1.

**Figure 5.**
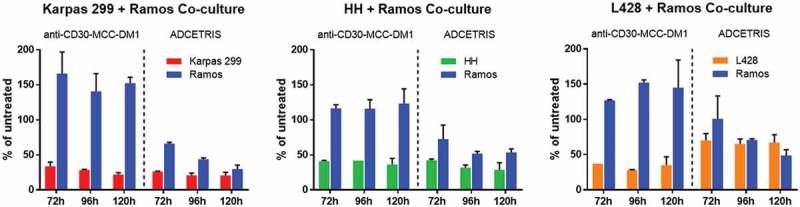
Bystander activities of ADCs. CD30-positive and CD30-negative cells were co-cultured in dual chamber Transwell dishes. The CD30-positive cells (Karpas 299 cells, HH cells and L428 cells) were plated into the upper Transwell chamber, and CD30-negative Ramos cells were plated into the lower Transwell chamber. Cocultures were either untreated or treated with anti-CD30-MCC-DM1 or ADCETRIS. Counting represents the number of surviving CD30-positive and CD30-negative cells following 72, 96, and 120 h treatment. Results for each test are plotted as the mean (± SEM).

The bystander effects of ADCETRIS have been shown in previous studies, but released Lys-MCC-DM1 is no longer active after leaving the target cells.^24^ We explored whether the anti-CD30-MCC-DM1 elicits bystander activity in cocultures with CD30-positive and CD30-negative cell lines. We used dual-chamber Transwell dishes to test whether medium from ADC-treated CD30-positive cells would induce bystander cell death in CD30-negative Ramos cells. Analysis of cocultures of HH/Ramos, Karpas 299/Ramos and L428/Ramos cells showed that treatment with 7 nmol/L ADCETRIS eliminated both populations of cells (Figure 5).

Similar treatment of these cell mixtures with 7 nmol/L anti-CD30-MCC-DM1 produced a different outcome, wherein only CD30-positive cells were killed while anti-CD30-MCC-DM1 displayed no potent reduction of CD30-negative cells. Interestingly, as the number of positive cells in the anti-CD30-MCC-DM1 treated co-culture group decreased, the number of negative cells was increased compared with their controls in the untreated coculture group, which suggests Lys-MCC-DM1 released from dead positive cells has no toxicity to cocultured negative cells. However, due to the decrease of positive cells, nutritional components remained in the coculture system, so that the negative cells could obtain more nutrition, while the positive and negative cells in the untreated coculture group competed to share the same medium.

In this experiment, L428 cells were more sensitive to anti-CD30-MCC-DM1 than ADCETRIS at the same dose of drug treatment. However, in the L428/Ramos co-culture group, ADCETRIS, but not CD30, resulted in the death of Ramos cells. These results showed that released Lys-MCC-DM1 from the cocultured CD30-positive cells exhibited no cytotoxic effect on CD30-negative cells. Thus, unlike ADCETRIS, anti-CD30-MCC-DM1 has no bystander activity on neighboring antigen-negative cells in culture.

### Efficacy of anti-cd30-mcc-dm1 in xenograft mouse models

Anti-CD30-MCC-DM1 was evaluated in preclinical models of CD30-positive lymphomas using the Karpas 299, HH, and L428 cell lines. Karpas 299 and HH cells were inoculated into CB17/SCID beige mice, and L428 cells were inoculated into NPG immunodeficient mice.

**Figure 6.**
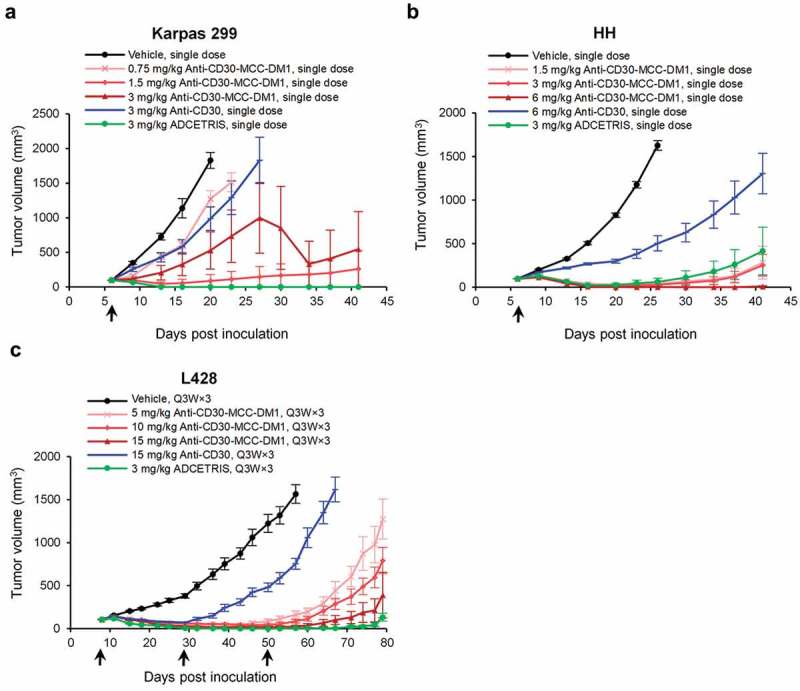
Efficacy of anti-CD30-MCC-DM1 in subcutaneous inoculation tumor xenograft models. Tumor growth is plotted as the mean (± SEM) tumor volume of each group receiving intravenous dose of vehicle, anti-CD30-MCC-DM1, anti-CD30, or ADCETRIS over the duration of the study. a, HH xenograft CB-17/SCID mice (*n* = 8 mice/group) with an average starting tumor volume of 98 mm^3^ were treated with a single dose as indicated. b, Karpas 299 xenograft CB-17/SCID mice (*n* = 8 mice/group) with an average starting tumor volume of 99 mm^3^ were treated with a single dose as indicated. c, L428 xenograft NPG mice (*n* = 8 mice/group) with an average starting tumor volume of 106 mm^3^ were treated with multiple doses as indicated.

Treatment of mice bearing subcutaneous lymphoma xenografts with a single dose or multiple doses of anti-CD30-MCC-DM1 resulted in significant tumor growth delay in a dose-dependent manner (Figure 6). At the same dose level, the antitumor activity of anti-CD30-MCC-DM1 was stronger than that of the anti-CD30 antibody control. In the HH model, the antitumor activity of anti-CD30-MCC-DM1 was slightly higher at 3 mg/kg compared with ADCETRIS at the same dose. Furthermore, at 6 mg/kg, mice achieved durable complete tumor regressions as defined by a lack of palpable tumor through the end of the experiment. In Karpas 299 and L428 models, a higher dose of anti-CD30-MCC-DM1 may be required to achieve the same tumor inhibition effect of ADCETRIS. In addition, the tumor volume of the anti-CD30-MCC-DM1 3 mg/kg group was highly variable in Karpas 299 models, presumably due to the limited number of mice per group (*n* = 8) and individual sensitivity differences, as five of eight tumors in this group disappeared completely, but the three other mice had poor sensitivity. We found that Karpas 299 transplanted tumor has a certain degree of variability, the tumor growth inhibitions of 3 mg/kg anti-CD30-MCC-DM1 were 71% (Figure 6(a)) and 92% (data are not shown) in two independent studies.

### Pharmacokinetics of anti-CD30-MCC-DM1 in monkeys

**Figure 7.**
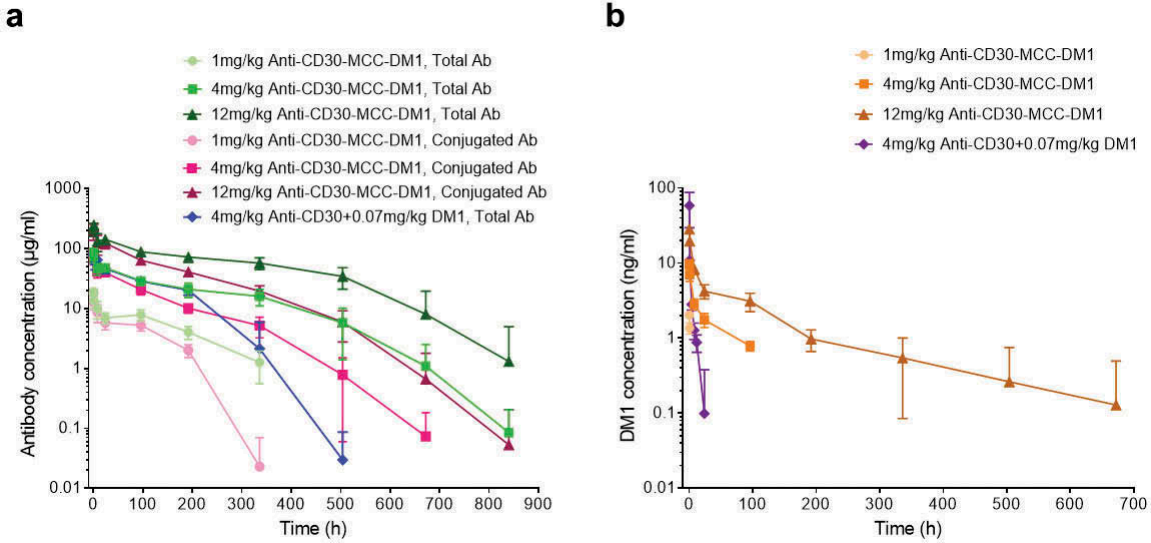
Pharmacokinetics of anti-CD30-MCC-DM1 in cynomolgus monkeys. Groups of cynomolgus monkeys (*n* = 8) were treated with a single dose of 1, 4, or 12 mg/kg anti-CD30-MCC-DM1 or 4 mg/kg anti-CD30 mAb mixed with 0.07 mg/kg DM1. Concentrations of total and conjugated antibody (a) as well as of DM1 (b) in plasma were analyzed by ELISA or LC-MS/MS and plotted as the mean (± SD).

After a single intravenous administration of anti-CD30-MCC-DM1, the plasma anti-CD30-MCC-DM1 concentrations decreased exponentially (Figure 7(a)). At different doses (1, 4, and 12 mg/kg) in cynomolgus monkeys, the peak concentrations and exposure levels of serum drugs (total antibody and conjugated antibody) increased with the increase in dosage, showing a nonlinear pharmacokinetic (PK) characteristic. At the same dose, the concentration difference between the total antibody and the conjugated antibody gradually increased with the extension of time, and the curves of each dose group were gradually separated. The ratio of total antibody to conjugate antibody was about 1.8 in each ADC group. It is suggested that the conjugated DM1 was deconjugated slowly after ADC drugs entered the body and the rates of drug deconjugation were consistent between different dosage groups.

At the end of the study (Day 35), anti-drug antibody (ADA) appeared in all groups, and occurred earlier (Day 14) in 1 mg/kg anti-CD30-MCC-DM1 and 4 mg/kg anti-CD30 + 0.07mg/kg DM1 groups in particular (Supplementary Table. S1). Consequently, the 4 mg/kg anti-CD30 mAb codosed with DM1 was cleared faster than 4 mg/kg anti-CD30-MCC-DM1. Because of the severe influence of ADA, the concentrations of total antibody and conjugated antibody in these two groups were not suitable for PK calculations. As shown in Figure 7(a), the concentrations of total antibody in 4 and 12 mg/kg anti-CD30-MCC-DM1 groups were not significantly affected by ADA before 504 h, and the conjugated antibody in these two groups were not significantly affected by ADA before 336 h. Therefore, the PK calculations of total antibody and conjugated antibody were based on the data from 0 to 504 h and 336 h, respectively. The half-life of total antibody in 4 mg/kg anti-CD30-MCC-DM1 treated monkeys was 7 days, while the half-life was close to 13 days in 12 mg/kg anti-CD30-MCC-DM1 group. The half-lives of conjugated antibodies in 4 and 12 mg/kg anti-CD30-MCC-DM1 groups were about 5 days (Table 2).

**Table 2.** Pharmacokinetics of anti-CD30-MCC-DM1 in monkeys.

	Total antibody of anti-CD30-MCC-DM1^a^		Conjugated antibody of anti-CD30-MCC-DM1^b^	
PK variables	4 mg/kg anti-CD30-MCC-DM1	12 mg/kg anti-CD30-MCC-DM1	4 mg/kg anti-CD30-MCC-DM1	12 mg/kg anti-CD30-MCC-DM1
t _1/2_ (hr)	171.73 ± 83.22	308.05 ± 166.02	115.23 ± 28.01	140.17 ± 24.15
C_max (_µg/mL)	87.55 ± 9.33	264.10 ± 22.90	85.47 ± 9.72	245.47 ± 23.72
AUC_(0- t)_ (hr*µg/mL)	10875.30 ± 2148.72	36922.09 ± 5251.25	5885.95 ± 862.45	19518.77 ± 2375.28
AUC_(0-inf)_ (hr*µg/mL)	12725.344 ± 3305.56	54904.93 ± 21938.43	6790.17 ± 1115.00	23609.28 ± 2965.47
Vd (mL/kg)	75.37 ± 23.87	94.91 ± 16.49	98.92 ± 24.10	103.43 ± 17.16
Cl (mL/hr/kg)	0.337 ± 0.101	0.242 ± 0.0715	0.604 ± 0.105	0.515 ± 0.0631
MRT_inf_ (hr)	251.26 ± 102.19	429.14 ± 232.13	150.66 ± 36.37	181.39 ± 36.04
	**DM1**			
PK variables	1 mg/kg anti-CD30-MCC-DM1	4 mg/kg anti-CD30-MCC-DM1	12 mg/kg anti-CD30-MCC-DM1	4 mg/kg anti-CD30 + 0.07mg/kg DM1
T_max_ (hr)	0.500 ± 0.000	0.500 ± 0.000	0.500 ± 0.000	0.500 ± 0.000
C_max_ (ng/mL)	2.04 ± 0.24	9.71 ± 1.36	28.40 ± 2.71	58.53 ± 28.89
AUC_(0-t)_ (hr*ng/mL)	1.36 ± 0.15	169.39 ± 27.21	828.15 ± 210.75	66.15 ± 16.87

^a^The time range for calculating the PK variables of the total antibody is 0–504 h.

^b^The time range for calculating the PK variables of the conjugated antibody is 0–336 h.

When analyzing the results of the DM1 concentration in plasma, we found that the exposure level increased nonlinearly with time (Figure 7(b)). After a single intravenous infusion of 4 mg/kg anti-CD30 mAb and 0.07 mg/kg DM1, the *C*_max_ and AUC_(0-t)_ of DM1 were 6.03 and 0.391 times those in the same DM1 equivalent of the anti-CD30-MCC-DM1 group, respectively. Our results suggested that the peak concentration of DM1 conjugated with antibody was significantly lower than that of DM1 simply mixed with antibody. Additionally, although the free DM1 is eliminated rapidly in the blood, the deconjugated DM1 could maintain longer cytotoxic time than codosed DM1 because of its slow shedding from the ADC.

### Safety profile of anti-CD30-MCC-DM1 in cynomolgus monkeys

Cynomolgus monkeys were intravenously administered a single dose of anti-CD30-MCC-DM1 or ADCETRIS. The dosage of ADCETRIS was set to 6 mg/kg, in view of the lethal dose (6 mg/kg) reported in its preclinical data. After considering the data for MCC-DM1-based ADCs in monkeys, we used a dose of 30 mg/kg to evaluate the safety of anti-CD30-MCC-DM1 in this study. The results showed that anti-CD30-MCC-DM1 was well tolerated with no death; however, 50% mortality occurred in the ADCETRIS-treated group (Table 3). Both animals were observed to experience abdominal pain combined with decreased activity and appetite before death, and the weights on day 13 decreased by 13% and 5% compared with that before administration. Histopathological examination showed that the death of the two animals was caused by drug toxicity, which was associated with liver, thymus (decreased immune function), and secondary pulmonary lesions as well as skin and mucosal lesions.

**Table 3.** Toxicity study of anti-CD30-MCC-DM1 and ADCETRIS in monkeys.

Drug	Anti-CD30-MCC-DM1	ADCETRIS
Dose (mg/kg)	30	6
No. of animals (Gender)	4 (2♂, 2♀)	4 (2♂, 2♀)
Mortality	0/4	2/4
Clinical observation		
Erythema	4/4	4/4
Papule	0/4	3/4
Loss of appetite	3/4	3/4
Necropsy		
Suppurative inflammation of shoulder skin	1/2	0/2
Inflammatory cell infiltration in the dermis	0/2	2/2

**Figure 8.**
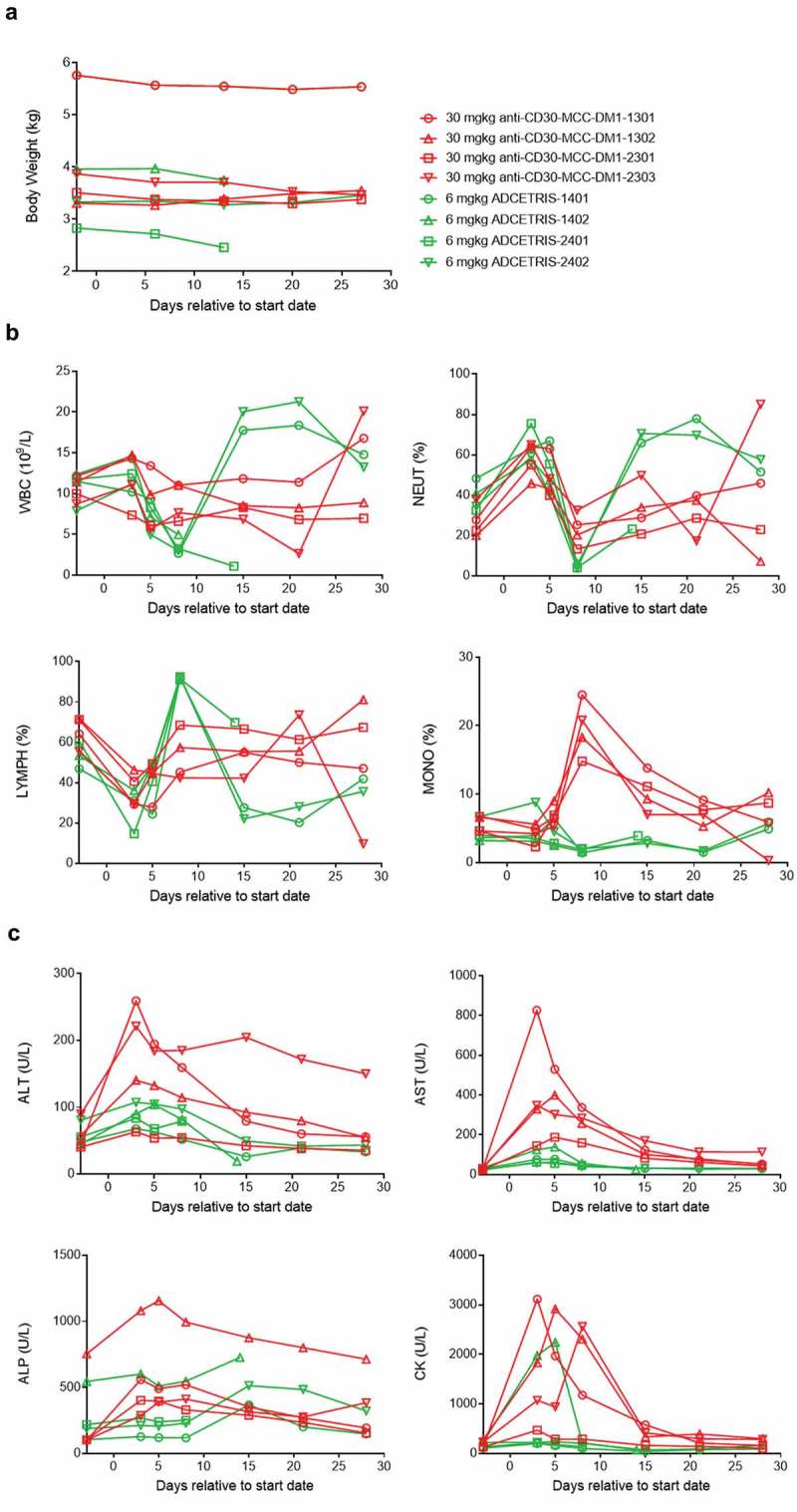
Effect of anti-CD30-MCC-DM1 and ADCETRIS on cynomolgus monkeys. Groups of cynomolgus monkeys (*n* = 4) were treated with a single dose of 30 mg/kg anti-CD30-MCC-DM1 or 6 mg/kg ADCETRIS. The effects of these drugs on body weight (a), selected hematology (b), and selected serum biochemistry (c) are presented with individual data. ALT: alanine aminotransferase, AST: aspartate aminotransferase, ALP: alkaline phosphatase, CK: creatine kinase, WBC: white blood cell count, NEUT: neutrophils, LYMPH: lymphocytes, MONO: monocytes.

Erythema was observed in all animals in both groups, and skin papules (3/4) were only observed in the ADCETRIS group. The body weights of the anti-CD30-MCC-DM1 group decreased significantly (3/4) and remained lower than before administration through 4 weeks after withdrawal, consistent with the loss of appetite (Figure 8(a)). The appetites and weights in the ADCETRIS group also decreased significantly before returning to normal after Day 18 in surviving animals (2/4).

The incidence of hematological changes was low in the anti-CD30-MCC-DM1 group; mainly, neutrophils decreased (3/4) and recovered after 7 days. In the ADCETRIS group, the lymphocytes increased from Day 5 to Day 8, while white blood cells, neutrophils and monocytes decreased significantly on Day 8 and recovered after 7 days (Figure 8(b)).

Serum biochemical changes were more obvious in the anti-CD30-MCC-DM1 group, including the observation that alanine aminotransferase (ALT), aspartate aminotransferase (ASP), alkaline phosphatase (ALP), and creatine kinase (CK) were elevated, peaked from Day 3 to Day 5, and returned to normal at Day 15. Serum biochemical changes in the ADCETRIS group were seen in only one monkey: AST and CK increased on Day 3 and Day 5, respectively (Figure 8(c)).

Necropsy revealed that one animal in the anti-CD30-MCC-DM1 group had a scab on the shoulder skin, and histopathological examination revealed purulent inflammation. Both monkeys in the ADCETRIS group had dark red spots on their skin affecting the chest, groin and limbs, which suggested inflammatory cell infiltration or inflammation with fibrous tissue hyperplasia in the dermis, as determined by pathological analysis.

## Discussion

The appropriate choices of mAb, drug potency, and conjugation methodology are key elements in developing optimized ADCs for the targeted treatment of cancer. Classical ADCs stably conjugated with highly potent drugs permit selective targeting of chemotherapeutic agents that would otherwise be too toxic for general administration. In the circulation, premature drug release could result in untoward toxicities and a reduction in the therapeutic efficacy of the ADCs. In recent years, new ADCs as potential treatments for diverse epithelial cancers have appeared. For example, sacituzumab govitecan (IMMU-132) incorporates a moderately toxic drug, SN-38, at a high ratio. It uses a pH-sensitive, cleavable linker designed to impart cytotoxic activity to both target and bystander cells via ADC internalization and local release of the free drug at the tumor.^25,26^

In this work, we redesigned anti-CD30-vcMMAE (ADCETRIS) to make it safer in the blood system by conjugating the antibody to DM1 through a noncleavable peptide linker to create anti-CD30-MCC-DM1. Both MMAE and DM1 belong to the class of tubulin inhibitors, and with free drug IC_50_ values between 10^−11^ and 10^−9^, they have been used successfully in commercialized ADCs.^27,28^ ADCETRIS uses a cleavable linker to release MMAE, increasing the possibility of the bystander effect. Anti-CD30-MCC-DM1, which uses a noncleavable linker, was found to release DM1 still attached to the linker in addition to an amino acid residue.

The targeting of CD30 has an important and emerging role in the setting of Hodgkin lymphoma, ALCL, and possibly other CD30-positive malignancies.^29^ CD30 has restricted expression in normal tissues, but is widely expressed in Hodgkin’s disease, anaplastic large cell lymphoma, non-Hodgkin’s lymphoma, multiple myeloma and cutaneous T-cell lymphoma.^21,30^ We showed that CD30-positive cell lines were sensitive to anti-CD30-MCC-DM1 cytotoxicity *in vitro*. Although the expression of CD30 was required for cells to be sensitive to anti-CD30-MCC-DM1, there was no direct correlation between antigen expression level and sensitivity. Except for L428, the potencies of anti-CD30-MCC-DM1 in these cells were comparable to that of ADCETRIS. L428 cells were sensitive to anti-CD30-MCC-DM1, whereas they were approximately 4 orders of magnitude less sensitive to ADCETRIS (data not shown). The reason for the L428 cell insensitivity to ADCETRIS *in vitro* remains to be explored. We also demonstrated that the binding and internalization of ADC in lymphoma cells with different CD30 expression levels were not affected by the conjugation of DM1. In contrast with ADCETRIS, which uses cleavable linkers that are reliant on distinctive intracellular conditions to release MMAE, anti-CD30-MCC-DM1 employing a noncleavable linker depends solely on the process of lysosomal degradation following ADC-antigen internalization. The concentrations of intracellular and extracellular Lys-MCC-DM1 were detectable after 12–24 h of treatment. As a consequence, the released drugs block microtubule assembly and arrest cells in G2/M phase of the cell cycle. It has previously been shown that the charged lysine adduct was much less active than the thioether derivative as a free drug.^31^ Therefore, in our results, it was not surprising that ADCETRIS elicited a strong bystander effect, whereas anti-CD30-MCC-DM1 did not.

ALCL, HL, and CTCL are ADCETRIS clinical research tumors. In xenograft mouse models, anti-CD30-MCC-DM1 can achieve significant cell death or even complete regression of tumors at well-tolerated doses similar to ADCETRIS in these solid tumor models. Although L428 was found to be insensitive to ADCETRIS in cytotoxicity assays, a higher dose of anti-CD30-MCC-DM1 was required to achieve the same tumor inhibition effect of ADCETRIS in L428 solid tumor models. It has been shown that, after intracellular release, free MMAE appeared extracellularly over time.^14^ By this mechanism, free toxin might reach the circulation and cause toxicity in bystander cells. It was speculated that the bystander effect elicited by ADCETRIS may help in increasing antitumor activity in subcutaneous inoculation tumor xenografts. In intravenous inoculation models, the released MMAE would instead spread rapidly into the blood system. This may not only weaken the bystander effect of tumor cells around the target cells, but is also potentially toxic to other normal blood cells. To assess the activity of anti-CD30-MCC-DM1 in conditions of hematologic malignancies, disseminated models are in development, in which median survival time is the main survey index.

The results of single dose PK studies on cynomolgus monkeys showed that anti-CD30-MCC-DM1 had a significant dose-exposure relationship. The half-lives of conjugated antibodies of anti-CD30-MCC-DM1 in 4 and 12 mg/kg groups were about 5 days, and the clearance rate decreased with increasing dose. The slope of the elimination phase became steeper in each dose group (the lower dose group was more obvious than the middle and high dose groups), suggesting that it might be related to the production of ADA in some individuals. Anti-CD30-MCC-DM1 showed linear and nonlinear PK characteristics in the single-dose PK of rats and cynomolgus monkeys, respectively, which were consistent with that of T-DM1 in the same dosage of rats and cynomolgus monkeys.^32^ Therefore, anti-CD30-MCC-DM1 was expected to exhibit promising druggability in the clinical setting.

In single-dose acute toxicity studies, the tolerance of cynomolgus monkeys in the 30 mg/kg anti-CD30-MCC-DM1 group was higher than that of the 6 mg/kg ADCETRIS group. Thus, 30 mg/kg was the tolerable dose of anti-CD30-MCC-DM1, which was comparable to that of T-DM1,^33^ and 6 mg/kg was the lethal dose of ADCETRIS, which was consistent with the mortality reported in the preclinical data of ADCETRIS.^34,35^ Administration of both ADCs was associated with reversible skin, hematological and liver function changes, but liver function changes were more obvious in the anti-CD30-MCC-DM1 group (ALT, AST, ALP, and CK), while hematological changes were more notable in the ADCETRIS group (white blood cell count, neutrophils, lymphocytes, monocytes). After 4 weeks of withdrawal, all monkeys recovered from the lesions. Thus, anti-CD30-MCC-DM1 may have more safety advantages than ADCETRIS. Together, the data presented here support the expectation that anti-CD30-MCC-DM1 may have clinically relevant efficacy in patients with HL, ALCL, and CTCL, who have cancer tissue with CD30 expression.

## Materials and methods

### Cells and reagents

CD30-positive and -negative cell lines were obtained from the following sources: HH （ATCC, CRL-2105）; SU-DHL-1 (ATCC, CRL-2955); Karpas 299 (Cobioer, CBP60271); L428 (Creative Bioarray, CSC-C0322); L540 (kindly provided by Institute of Medicinal Biotechnology, Chinese Academy of Medical Sciences); Raji (Cell bank of typical culture preservation, Committee of Chinese Academy of Sciences, TCHu 44); and Ramos (Cell Resource Center, IBMS, CAMS/PUMC, 3111C0001CCC000047). These cell lines were cultured at 37°C with 5% CO_2_ in RPMI 1640 supplemented with 10% fetal bovine serum. ADCETRIS was from Seattle Genetics, Inc. (Bothell, WA). The anti-CD30 mAb was generated by Shanghai Fudan-Zhangjiang Bio-Pharmaceutical Co, Ltd. (Shanghai, China). DM1 and T-DM1 were provided by Shanghai Jiaolian Drug Research and Development Co., Ltd. (Shanghai, China).

### Preparation of anti-CD30-MCC-DM1

Purified anti-CD30 antibody was buffer exchanged into a solution containing 50 mM potassium phosphate, 50 mM sodium chloride and 2 mM EDTA, pH 7.4. SMCC (BrightGene, CAS 64987–85-5) was dissolved in dimethylacetamide (DMA) and added to the antibody solution to generate a final SMCC/antibody molar ratio of 7:1. The reaction was allowed to proceed for 15–20 min at room temperature (RT) with mixing. The MCC-modified antibody was subsequently purified with a HiTrap desalting column (G-25; GE, 17003301),which is equilibrated in a buffer containing 25 mM sodium citrate, 150 mM NaCl and 2 mM EDTA, pH 6.0. DM1, dissolved in DMA, was added to the purified MCC-modified antibody preparation to provide a molar ratio of 6:1 (DM1-to-mab ratio). The reaction was allowed to proceed for 1–2 h at RT with mixing. The DM1-modified antibody solution was diafiltered with 12 volumes of phosphate-buffered saline (PBS) to remove unreacted DM1, sterile-filtered, and stored at 4°C. Typically, an 80%–90% yield of antibody was achieved through this process.

The number of conjugated linker-drug molecules per mAb was calculated from the integrated peaks of the DAR species resolved by iCIEF (Maurice; Protein Simple, CA, USA). The DAR was confirmed by analyzing the intact ADC by LC-MS (Vion ESI Q-TOF; Waters, MA, USA) using polyphenyl reversed-phase column (450A, 2.7 μm; Waters, 186008945). Molecular masses were derived from multiply charged ions deconvoluted using UNIFI software (Waters).

### Determination of cytotoxicity and antigen level

Cytotoxicity was measured using the Alamar Blue (Invitrogen, Thermo Fisher Scientific, DAL1100) dye reduction assay according to the manufacturer’s directions. Briefly, 77 h after drug exposure, Alamar Blue solution was added to cells to constitute 15% culture volume. Cells were incubated for 19 h, and the fluorescence was measured on a SpectraMax M2^e^ multimode microplate reader (Molecular Devices, USA) using an excitation wavelength between 530 nm and an emission wavelength at 590 nm with SoftMax Pro 5.4.5 analysis software (Molecular Devices, USA).

Surface CD30 expression of different cell lines was quantified by using DAKO QIFIKIT (Agilent Technologies, K0078) and analyzed through flow cytometry. Briefly, 1 × 10^6^ cells were saturated with human CD30/TNFRSF8 antibody (R&D Systems, MAB229) for 60 min on ice while using the isotype control antibody for cell background. After primary incubation of the mAb, cells were washed twice with cold PBS and resuspended in 100 μl of QIFIKIT FITC secondary antibody diluted 1:50 with PBS. Secondary detection antibody incubation was conducted for 45 min at 4°C, protected from light. Then, cells were washed once and resuspended in PBS for flow cytometric analysis performed on an Attune NxT Flow Cytometer (Thermo Fisher Scientific, USA). Using the setup beads and calibration beads provided in the QIFIKIT, a standard curve was plotted using the median fluorescence intensity (MFI) against antibody-binding capacity (ABC). Subsequent extrapolation of the sample MFI values into a standard curve on the basis of the calibration bead MFIs was performed to calculate the number of antigens per cell.

### Multistep process of anti-CD30-MCC-DM1 in vitro

The effect of drug conjugation on antigen binding was evaluated by the cell-binding assay in a competition format. The chimeric anti-CD30 mAb was biotinylated using EZ-Link® Sulfo-NHS-LC-Biotin (Thermo Scientific, 21327). Varying concentrations of anti-CD30-MCC-DM1 and 0.33 nmol/L biotin-anti-CD30 mAb were coincubated with 5 × 10^5^ cells for 0.5 h at 4°C. The cells were washed with PBS to remove unbound agents, and were then stained with Avidin, NeutrAvidin™, and PE conjugate (Invitrogen, Thermo Fisher Scientific, A2660) as a secondary antibody for 0.5 h at 4°C. Cells incubated with biotin-anti-CD30 mAb in the absence of drugs served as controls. MFI values were measured using a FACSCalibur flow cytometer (BD Biosciences, USA). For data analysis, the percentage competition was calculated as follows: percentage competition (%) = (MFI control – MFI drug)/MFI control ×100. The dose response curve was fitted with a 4-parameter model using GraphPad Prism 7 software.

To measure the CD30 internalization after antibody and ADC binding, anti-CD30 mAb, and anti-CD30-MCC-DM1 were conjugated with pHAb dye (Promega, G9835) using thiol chemistry. Cells were plated in a 96-well black plate (Costar, Corning, 3603) at a density of 5 × 10^5^ cells/well. The pHAb-conjugated drugs were added to cells at the desired concentrations, mixed gently for 10 min on a plate mixer, and then incubated for 48 h to allow internalization. During the 48 h, plates were read on a fluorescent plate reader at Ex/Em: 532 nm/560 nm on an MD SpectraMax M2^e^ plate reader. Control wells were treated with only RPMI 1640 containing 10% fetal bovine serum, and the fluorescence signal from the cells alone, subtracted from the signal of positive wells, was used as background.

The contents of intracellular and extracellular released drug were determined with the LC-MS/MS method.^36^ ADC was added (400 ng/mL) to cells at a density of 5 × 10^5^ cells/mL, and the cultures were incubated at 37°C for up to 72 h. For intracellular drug quantitation, 150 μL of RIPA Lysis and Extraction Buffer (Thermo, 89900) was added to cell pellets and sonicated for 15 s, then incubated at 4°C, 300 rpm for 30 min. Two volumes of internal standard solution (AP-3 in 80% acetonitrile) was added and stored at −20°C for 30 min. For released drug quantitation in the culture medium, two volumes of internal standard solution were added to the supernatant and stored at −20°C for 30 min. All samples were centrifuged (14,000 × g, 30 min, 4°C) to remove protein before LC/MS quantitation. To derive an equation for the quantitation of drug content in the unknown samples, the peak area for each drug standard was divided by the peak area obtained for the internal standard. The resultant peak area ratios were plotted as a function of the standard concentrations, and the data points were fitted to a curve using linear regression. The peak area ratios obtained were converted to drug concentration using the derived equation.

To examine the cell cycle effects of anti-CD30-MCC-DM1, cells were cultured in complete media with or without a saturating level (7 nmol/L) of anti-CD30 mAb, anti-CD30-MCC-DM1, T-DM1 or equivalent molar concentration of DM1 (23 nmol/L). At 0, 16, 24, and 48 h after exposure, cells were fixed in 70% cold ethanol and stored at −20°C overnight. The cells were incubated in 50 μg/ml propidium iodide (Life Technologies, Thermo Fisher Scientific, P1304MP) with 0.4% RNase A (Takara, 2158) at RT in the dark for 30 min. The cell cycle analyses were performed on a FACSCalibur flow cytometer, and the data were analyzed using CellQuest software.

The bystander activity was assessed by coculture of CD30-positive and CD30-negative cells with dual chamber Transwell dishes. A total of 1.25 × 10^5^ CD30-negative cells were plated in the bottom chamber of the 6-well cell culture plates (Corning, CNG3472), while 1.25 × 10^5^ CD30-positive cells were plated in the upper Transwell permeable inserts, with a membrane pore-size of 0.4 μm, that fit into the 6-well cell culture plates. In this model, any potential bystander factors can be diffused through the permeable membrane. After 72–120 h of incubation with ADCs or medium control, the cell inserts in the upper and bottom wells were counted using a Countess automated cell counter (Invitrogen, Thermo Fisher Scientific, USA).

### Assessment of anti-CD30-MCC-DM1 in mice

All animal experiments were conducted in a facility accredited by the Association for Assessment of Laboratory Animal Care (AALAC) under institutional animal care and use committee (IACUC) guidelines and appropriate animal research approval. For the subcutaneous model, 5 × 10^6^ Karpas 299 or 1 × 10^7^ HH cells were injected in the flank of female CB-17/SCID beige mice, and 1 × 10^7^ L428 cells were injected in the flank of female NPG immunodeficient mice. Once tumors reached approximately 100 mm^3^, mice were treated with vehicle, antibody or ADCs (*n* = 8 mice/group). For single-dose administration, the dose of anti-CD30-MCC-DM1 for Karpas 299 was 0.75, 1.5, or 3mg/kg, respectively; the doses of anti-CD30 or ADCETRIS for Karpas 299 was 3mg/kg. The dose of anti-CD30-MCC-DM1 for HH was 1.5, 3, or 6mg/kg, respectively; the dose of anti-CD30 for HH was 6 mg/kg; the dose of ADCETRIS for HH was 3 mg/kg. For multidose administration (Q3W×3), the dose of anti-CD30-MCC-DM1 for L428 was 5, 10, or 15mg/kg, separately; the dose of anti-CD30 for L428 was 15 mg/kg; the dose of ADCETRIS for L428 was 3 mg/kg. Tumor volumes were measured twice per week.

### Pharmacokinetics of anti-CD30-MCC-DM1 in cynomolgus monkeys

Groups of cynomolgus monkeys (*n* = 8, 4, females and 4 males) were treated with a single dose of 1, 4, or 12 mg/kg anti-CD30-MCC-DM1 or 4 mg/kg anti-CD30 mAb mixed with 0.07 mg/kg DM1. Serum samples for conjugated and total anti-CD30-MCC-DM1 antibody measurements were collected up to 840 h after dosing, and plasma samples for DM1 determination were prepared up to 24 h postdose. Concentrations of conjugated and total anti-CD30-MCC-DM1 antibodies in plasma were determined with a validated ligand-binding fluorescence immunoassay; the lower limit of quantitation was 39.06 ng/mL. Concentrations of DM1 in plasma were determined with a validated LC-MS/MS method; the lower limit of quantitation was 0.500 ng/mL.

### Toxicity studies in cynomolgus monkeys

A single dose of 30 mg/kg anti-CD30-MCC-DM1 or 6 mg/kg ADCETRIS was intravenously administered to cynomolgus monkeys (*n* = 4, 2, females and 2 males). Clinical signs, body weight, food consumption, and clinical pathology were monitored throughout the study. The reversibility of the toxicity changes was assessed in a subsequent 4-week recovery period. A necropsy was conducted on the day after the recovery period (Day 29).

### Statistical analysis

All statistical analyses in mice were performed using SPSS 18.0. The survival curves of each group in the disseminated model were plotted using the Kaplan-Meier method, and log-rank tests were used to compare the survival differences among different groups. In PK studies, Watson LIMS^TM^ v.7.3.0.01 (Thermo Fisher Scientific, Inc.) software was used to manage all data, and the non-compartment model of WinNonLin® version 6.4 (Certara Corp.) software was used to analyze the PK parameters. In toxicity studies, for all numerical data (body weight and clinical pathology), the means and standard deviations of the groups were calculated. Statistics between groups were conducted using the Provantis 9.4.3 form and data statistics module.

## Abbreviations

ABC: antibody-binding capacity
ADA: anti-drug antibody
ADC: antibody-drug conjugate
ALCL: anaplastic large cell lymphoma
ALP: alkaline phosphatase
ALT: alanine aminotransferase
AST: aspartate aminotransferase
ATCC: American Type Culture Collection
CK: creatine kinase
CTCL: cutaneous T-cell lymphoma
DM1: maytansinoid *N*(2ʹ)-deacetyl-*N*(2ʹ)-(3-mercapto-1-oxopropyl)-maytansine
FDA: Food and Drug Administration
HL: Hodgkin’s lymphoma
iCIEF: imaged capillary isoelectric focusing
DAR: drug to antibody ratio
LC/MS: liquid chromatography-tandem mass spectrometry
LLOQ: lower limit of quantitation
LYMPH: lymphocytes
mAb: monoclonal antibody
MFI: median fluorescence intensity
MMAE: monomethyl auristatin E
MONO: monocytes
NEUT: neutrophils
SMCC: succinimidyl trans-4-(maleimidylmethyl)cyclohexane-1-carboxylate
T-DM1: Trastuzumab-DM1
WBC: white blood cell count
