# Supplementary material for: Conjugation of DM1 to anti-CD30 antibody has potential antitumor activity in CD30-positive hematological malignancies with lower systemic toxicity
Source: MAbs. 2019 Jun 4;11(6):1149–61. doi: 10.1080/19420862.2019.1618674 (PMC6748589; doi:10.1080/19420862.2019.1618674)
Supplement: Supplemental Material [file kmab-11-06-1618674-s001.docx]

Supplemental Material to:

**Conjugation of DM1 to anti-CD30 antibody has potential antitumor activity in CD30-positive hematological malignancies with lower systemic toxicity**

Yijun Shen^a,b*^, Tong Yang^b*^, Xuemei Cao^b^, Yifan Zhang^b^, Li Zhao^b^, Hua Li^b^, Teng Zhao^b^, Jun Xu^b^, Hengbin Zhang^b^, Qingsong Guo^b^, Junli Cai^b^, Bei Gao^b^, Helin Yu^b^, Sicheng Yin^b^, Ruiwen Song^b^, Jingsong Wu^b^, Lingyu Guan^b^, Guanghao Wu^c^, Li Jin^a^, Yong Su^b^, Yanjun Liu^c^

^a^Ministry of Education Key Laboratory of Contemporary Anthropology, Fudan University, Shanghai, China

^b^R&D Department of Genetic Engineering, Shanghai Fudan-Zhangjiang Bio-Pharmaceutical Co., Ltd., Shanghai, China

^c^Shanghai Jiaolian Drug Research and Development Co., Ltd. , Shanghai, China

**Contact** 79812251@qq.com

^*^These authors contributed equally to this work.

**
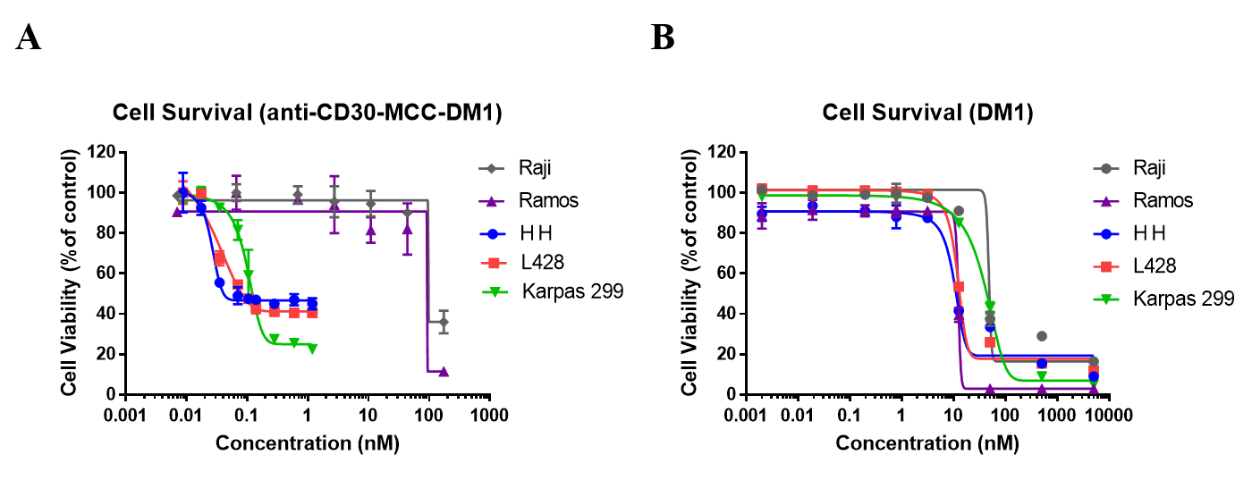
**

**Figure S1.** Cytotoxicity of anti-CD30-MCC-DM1 or DM1 on lymphoma lines. CD30-positive (HH, L428 and Karpas 299) and negative cells (Raji and Ramos) were plated at 5000 cells/well and were exposed to a gradient titration of anti-CD30-MCC-DM1 (DM1 equivalent molar concentration) or DM1. Cells were assessed for cytotoxicity by the Alamar Blue assay after 96 h of continuous exposure. The percentage cell viability was relative to untreated control wells. Results for each study are plotted as the mean (± SEM).

**
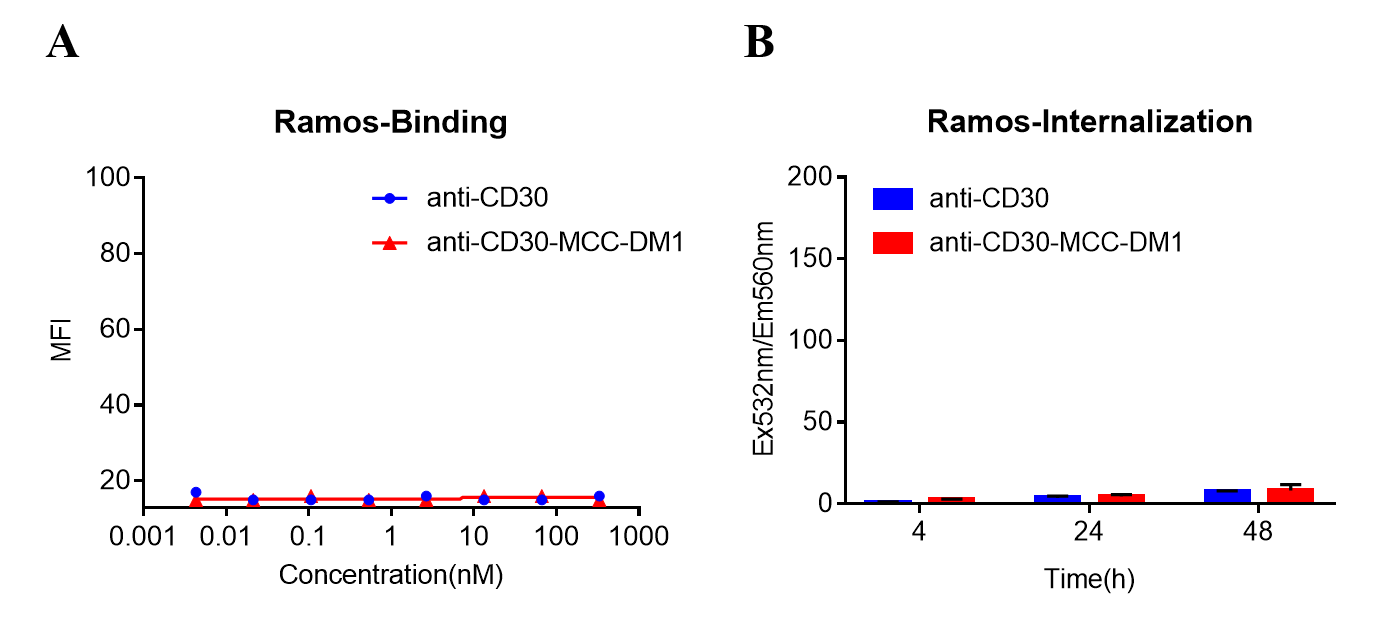
**

**Figure S2**. Binding and internalization data of CD30-negative Ramos cells. A, Competition binding of anti-CD30-MCC-DM1. Ramos cells were combined with biotinylated anti-CD30 and serial dilutions of either anti-CD30 or anti-CD30-MCC-DM1. The median fluorescence intensities were plotted versus mAb concentration as described in the “Materials and Methods”. B, internalization of anti-CD30-MCC-DM1 into Ramos cells. pHAb dye-conjugated anti-CD30 and anti-CD30-MCC-DM1 were added to the Ramos cells and incubated for various time durations. Mean and standard deviations from triplicate readings are plotted.

**Table S1.** Summary of ADA incidence in PK study of anti-CD30-MCC-DM1 in monkeys.

|  | Incidence of ADA | | | | Total Incidence of ADA |
| --- | --- | --- | --- | --- | --- |
|  | Day 0 | Day 14 | Day 21 | Day 35 |  |
| 1 mg/kg anti-CD30-MCC-DM1 | 0/8 | 7/8 | 8/8 | / | 8/8 |
| 4 mg/kg anti-CD30-MCC-DM1 | 0/8 | / | 4/8 | 6/8 | 6/8 |
| 12 mg/kg anti-CD30-MCC-DM1 | 1/8 | / | 1/8 | 7/8 | 7/8 |
| 4 mg/kg anti-CD30+0.07mg/kg DM1 | 0/8 | 5/8 | 7/8 | / | 7/8 |

“/” representative undetected
